# Nocturnal sleep mediates the relationship between morningness–eveningness preference and the sleep architecture of afternoon naps in university students

**DOI:** 10.1371/journal.pone.0185616

**Published:** 2017-10-17

**Authors:** Tzu-Yin Lee, Pi-Chen Chang, Ing-Jy Tseng, Min-Huey Chung

**Affiliations:** 1 School of Nursing, College of Nursing, Taipei Medical University, Taipei, Taiwan; 2 School of Gerontology Health Management, College of Nursing, Taipei Medical University, Taipei, Taiwan; 3 Department of Nursing, Taipei Medical University-Shuang Ho Hospital, New Taipei City, Taiwan; Associazione OASI Maria SS, ITALY

## Abstract

The present study investigated the parameters of nocturnal sleep that mediate the relationship between morningness–eveningness preference and the sleep architecture of naps in university students. This study had a cross-sectional, descriptive correlational design. The sleep architecture of 52 students invited to take an afternoon nap in the laboratory was recorded. The morningness–eveningness questionnaire (MEQ) was used to evaluate morningness–eveningness preference. An actigraph was used to collect students’ nighttime sleep data in the week preceding the study. Polysomnography was used to measure the sleep architecture of the participants’ naps. After adjustments for potential factors, although the MEQ did not directly correlate with the percentage of sleep stages in naps, the effects of the MEQ on the percentage of Stage 1 sleep, slow-wave sleep, and rapid eye movement sleep; sleep duration; and sleep efficiency of naps were mediated by the total sleep time in the preceding week. This preliminary study suggests that nap quality was affected by morningness–eveningness preference through the mediation of total nocturnal sleep time. Therefore, future studies should be carefully designed to consider nighttime sleep patterns when analyzing the effects of chronotypes on daytime sleep.

## Introduction

Napping refers to a short duration of sleep during the day. A decline in arousal after half a day of activity may compel people to take a break [1]. A 30-min nap was reported to improve participants’ reaction times [2]. Increasing evidence has revealed the physiological and psychological benefits of napping [3–5]. A chronotype is one of the most crucial differences in human circadian preference; it can be defined in terms of the propensity of an individual to engage in activity and sleep at particular times during a 24-h period [6]. “Eveningness” and “morningness” are the two extremes [7]. Studies have extensively examined the association of chronotypes with daytime sleepiness [8, 9], napping behavior [10, 11], and sleep latency during naps [12]. However, data on the possible factors mediating the relationship between chronotypes and the sleep architecture of naps remain scarce. A thorough understanding of the relationship between circadian preference, nocturnal sleep, and daytime sleep could help researchers to control the interaction effect when investigating sleep-related behaviors and gain a comprehensive understanding of individual sleep tendencies when managing patients’ sleep disorders.

In contrast to napping, nocturnal sleep is the main sleep period influenced by a person’s circadian rhythm, which significantly correlates with nap quality [13–15]. More sleep fragmentation during nighttime sleep was associated with a higher risk of napping but not nap duration [13]. Significant correlations were observed between weekend nighttime sleep and weekday perceived sleep debt and nap length [14]. Participants who napped for more than 2 h had poorer nighttime sleep quality [15]. Therefore, individual differences in the tendency to nap could be a consequence of disturbed or decreased nighttime sleep.

A chronotype strongly correlates with the quality of nighttime sleep. One study indicated that chronotypes more comprehensively explain individual differences in nocturnal sleep than does age [16]. Children of evening type had shorter nocturnal sleep times during weekdays than did other children [17]. Adolescents with eveningness are more likely to have poorer sleep quality and sleep less on weekdays than those with morningness [10, 18]. Adults of morning type had higher sleep efficiency than those of evening type [19]. Compared with participants of morning and intermediate type, those of evening type have reported more time in bed and greater variability in time out of bed [9, 20]. Thus, the relationship between morningness–eveningness preference and the quality and quantity of nighttime sleep has been confirmed.

Studies have reported the association between morningness–eveningness preference and nocturnal sleep [20–23], as well as that between nighttime and daytime sleep [13–15], and have indicated that evening-type naps during weekdays are more frequent than morning-type naps [10]. However, few studies have examined the association between morningness–eveningness and the sleep latency of naps [12]. Moreover, people with eveningness have higher sleep debt and therefore suffer from daytime sleepiness; their high sleep tendencies during daytime is a concern [8]. Additional studies are warranted to obtain the details of nocturnal sleep among participants to comprehensively understand the association between morningness–eveningness preference and the sleep architecture of naps. Future studies must determine whether the sleep stages of naps are affected by nocturnal sleep across different chronotypes. Therefore, this study investigated the parameters of nocturnal sleep that mediate the relationship between morningness–eveningness preference and the sleep architecture of naps in university students.

## Materials and methods

### Study design and participants

This study had a cross-sectional, descriptive correlational design. The study participants were university students who fulfilled the following criteria: (1) age, 18–23 years; (2) no sleep-related diseases reported in the self-administered questionnaire; (3) not diagnosed with heart disease, acute myocardial infarction, arrhythmia, or other cardiovascular diseases; and (4) not taking antidepressants or psychotropic or cardiovascular-related medicines such as antihypertensive, hypoglycemic, and antihyperlipidemic agents. The required sample size was estimated using G*power 3.1 [24]. The effect size was set at .35 with an alpha value of .05. The number of predictors was set at 5 and the power set was at .95. The estimated sample size for linear multiple regression was 48 participants.

### Measures

#### Morningness–eveningness questionnaire

The morningness–eveningness questionnaire (MEQ), a self-reported questionnaire, assesses a person’s chronotype [25] and comprises 19 items associated with the respondent’s preferred times for rising and retiring, physical state, and mental performance. Participants can be classified into five categories: definite and moderate evening-type sleep, neither-type sleep, and moderate and definite morning-type sleep groups. The MEQ scores range from 16 to 86, with higher scores reflecting higher morningness preference. In this study, the sample was split by median MEQ score, forming Morning Tendency group and Non-Morning Tendency group [26]. The Chinese version of the MEQ had a reliability coefficient of 0.769 [27].

#### Actigraph

The participants were asked to wear a wrist actigraph (Mini Motionlogger Actigraphy, Ambulatory Monitoring, Ardsley, NY, USA) for 7 consecutive days before taking a nap in the laboratory to gain an objective measure of nocturnal sleep. They also kept a sleep diary for a week to record their bedtimes and rise times, which were used to analyze the actigraph data. The participants’ daily activities were recorded in the zero-crossing method mode and analyzed using Action W2 (Ambulatory Monitoring). The outcome data used in this study included the mean total sleep time (TST), sleep onset latency (SOL), and wake time after sleep onset (WASO).

#### Polysomnography

Polysomnography (PSG) is a multichannel recording technique that can monitor various bodily functions during sleep, including brain waves, eye movement, muscle activity or skeletal muscle activation, and heart rhythm. The participants were connected to a Covidien Sandman PSG monitor (Ottawa, Canada) by using four electrode points (locations C3, C4, O1, and O2) and two reference points (locations A1 and A2). The electrode impedance was set to ≤5 and the rate of recording was 200 times per s. To analyze the sleep stages in this study, PSG data were imported into sleep analysis software (Somnologica 3.1.2, Embla, USA) and analyzed based on the criteria reported by Rechtschaffen and Kales [28]. The sleep architecture of naps was divided into four stages, namely stages 1 and 2, the slow-wave sleep (SWS) stage, and the rapid eye movement (REM) stage. Sleep duration, SOL, WASO, and sleep efficiency were also recorded.

### Data collection

Each participant was administered the MEQ and an actigraph to collect their sleep data. Moreover, PSG data were used to measure the sleep architecture of their naps. On the study day, the participants were free to start the nap test in our laboratory between 13:00 and 15:00. Sleep technicians first introduced the setting and environment. After the placement of patches, the participants began to nap. Light and sound disturbances were avoided in the laboratory during the nap time. No maximum nap time was set and the participants could end their naps according to their preferences. All participants fell asleep successfully, indicating that the duration of all experienced sleep stages exceeded 30 s.

### Ethical considerations

Ethical approval for this experiment was obtained from the Joint Institutional Review Board of Taipei Medical University (approval number: 200910002). Participation in this study was voluntary and anonymous. Before the study, all participants signed the informed consent form, which explained the study and informed them of their responsibilities and rights to withdraw at any time during the study.

### Statistical analysis

The demographic factors in this study were sex; body mass index (BMI); caffeine consumption habits; nap habits; mean TST, SOL, and WASO in the week preceding the study; and MEQ score. Nap and caffeine consumption habits were categorized into two groups: zero or one or more times per week. Based on the overweight criteria used in Taiwan [29], the participants were classified as BMI ≥ 24 and BMI < 24. Differences in the baseline characteristics of PSG-recorded sleep parameters were analyzed using a *t* test or Pearson correlation. The SWS and REM stages in the PSG-recorded data reached 0 for several participants; therefore, we used Box–Cox transformations with λ_1_ = 0 and λ_2_ = 0.01 before conducting analyses [30].

To determine whether TST, SOL, and WASO mediate the relationship between the MEQ and napping, regression analyses were conducted based on the guidelines proposed by Shrout and Bolger [31]. Furthermore, to confirm the relevance of the indirect effects, a nonparametric bootstrapping procedure was performed [32]. A total of 1000 bootstrap resamples were created to obtain bias-corrected estimates. Analyses were performed using SPSS Version 19.0 for Windows (SPSS, Inc., Chicago, IL, USA). All statistical tests were two-tailed and *p* < 0.05 was considered statistically significant.

## Results

Our study examined 52 university students: 7 men (13.5%) and 45 women (86.5%). Table 1 shows the participants’ demographic data, namely their BMI (≥24 for 19.2% of the participants), caffeine consumption habits (84.6%), and naps habits (82.7%). The mean bedtime and rise time in the week preceding the study were 1:25 and 8:34, respectively. The mean TST, SOL, and WASO recorded using the actigraph were 409.9, 19.2, and 19.9 min, respectively. The mean efficiency of nocturnal sleep in the preceding week was 95.1%. The participants’ circadian preferences were classified into two groups: morning tendency (n = 26; 50.0%) and non-morning tendency (n = 26; 50.0%).

**Table 1:** Demographic characteristics of the participants (N = 52).

	Total participants
Number of participants	52
Sex (n [%])	
Male	7 (13.5)
Female	45 (86.5)
BMI (n [%])	
<24	42 (80.8)
≥24	10 (19.2)
Caffeine consumption habits (n [%])	
No	8 (15.4)
Yes	44 (84.6)
Nap habits (n [%])	
No	9 (17.3)
Yes	43 (82.7)
Prior week (mean [SD])	
Bedtime (hh:min)	1:25 (1:07)
Rise time (hh:min)	8:34 (1:15)
TST (min)	409.9 (51.1)
SOL (min)	19.2 (15.1)
WASO (times)	19.9 (18.2)
Sleep efficiency (%)	95.1 (4.9)
Circadian preference (n [%])	
Morning tendency	26 (50.0)
Non-Morning tendency	26 (50.0)

BMI, body mass index; TST, total sleep time; SOL, sleep onset latency; WASO, wake time after sleep onset.

Table 2 shows the association between demographic and PSG-recorded nap data. The mean sleep duration for naps was 59.5 min (standard deviation [SD]: 34.0). The mean percentages of Stages 1 and 2 sleep were 27.8% (SD: 17.1) and 53.9% (SD: 17.0), respectively. The mean percentages of SWS and REM sleep after Box-Cox transformation were 0.1 (SD: 3.7) and −2.6 (SD: 3.2), respectively. No significant differences were observed in PSG-recorded naps between male and female participants, in caffeine consumption habits, or in nap habits. However, participants with BMI ≥ 24 were found to have shorter sleep durations (*p* < 0.05), lower percentages of REM sleep (*p* < 0.01), and lower sleep efficiency (*p* < 0.05) of naps. The Pearson correlation test revealed that the participants with longer TSTs in the preceding week had a significantly higher Stage 1% (*p* < 0.05), shorter SWS% (*p* < 0.01), shorter REM% (*p* < 0.01), shorter sleep durations (*p* < 0.05), and lower sleep efficiency (*p* < 0.01) of naps. Significant correlations were observed between the MEQ score and REM% (*p* < 0.05), sleep duration (*p* < 0.05), and percentage of sleep efficiency (*p* < 0.05) of naps.

**Table 2:** Results of *t* test and Pearson correlation for demographic characteristics and PSG-recorded naps (N = 52).

	Afternoon nap					
	Stage 1 (%)	Stage 2 (%)	SWS (%)^+^	REM (%)^+^	Sleep duration (min)	Sleep efficiency (%)
Total participants	27.8 (17.1)	53.9 (17.0)	0.1 (3.7)	−2.6 (3.2)	59.5 (34.0)	70.5 (19.0)
Sex (mean [SD])						
Male	37.8 (23.8)	46.7 (13.8)	−0.3(4.0)	−2.5 (3.6)	50.5 (33.7)	62.8 (25.0)
Female	26.3 (15.6)	55.0 (17.4)	0.1(3.7)	−2.6 (3.2)	60.9 (34.3)	71.1 (17.9)
*p* value	0.26	0.23	0.78	0.94	0.46	0.25
BMI (mean [SD])						
<24	26.2 (16.0)	53.3 (15.9)	0.3 (3.6)	−2.1 (3.4)	64.1 (35.0)	73.8 (17.8)
≥24	34.7 (20.4)	56.4 (22.2)	−1.3 (3.7)	−4.6 (0.0)	40.1 (21.6)	56.8 (18.4)
*p* value	0.16	0.62	0.20	<0.01	<0.05	<0.05
Caffeine consumption habits (mean [SD])						
No	30.6 (16.5)	54.6 (18.0)	−0.1 (3.8)	−2.9 (3.2)	66.9 (37.9)	71.6 (18.7)
Yes	27.3 (17.3)	52.7 (17.1)	0.1 (3.7)	−2.5 (3.3)	58.1 (33.6)	70.3 (19.2)
*p* value	0.62	0.90	0.93	0.79	0.51	0.87
Nap habits (mean [SD])						
No	22.7 (10.4)	60.5 (19.1)	0.8 (3.3)	−3.0 (3.2)	51.3 (17.6)	69.7 (16.7)
Yes	28.9 (18.1)	52.5 (16.5)	−0.2 (3.8)	−2.5 (3.3)	61.2 (36.5)	70.7 (19.6)
*p* value	0.33	0.20	0.44	0.66	0.43	0.89
	*r*	*r*	*r*	*r*	*r*	*r*
MEQ score	0.09	0.17	−0.02	−0.30*	−0.28*	−0.32*
TST in the preceding week	0.35*	0.20	−0.44**	−0.36**	−0.36*	−0.40**
SOL in the preceding week	0.05	−0.11	−0.09	0.06	−0.01	0.09
WASO in the preceding week	0.18	−0.08	−0.04	−0.03	−0.08	0.03
Sleep efficiency in the preceding week	−0.17	0.08	0.05	0.01	0.05	−0.06

^+ ^Box–Cox transformations with λ_1_ = 0 and λ_2_ = 0.01.

* *p* < 0.05.

** *p* < 0.01.

MEQ, morningness–eveningness questionnaire; TST, total sleep time; SOL, sleep onset latency; WASO, wake time after sleep onset; SWS, slow-wave sleep REM, rapid eye movement.

**Fig 1:**
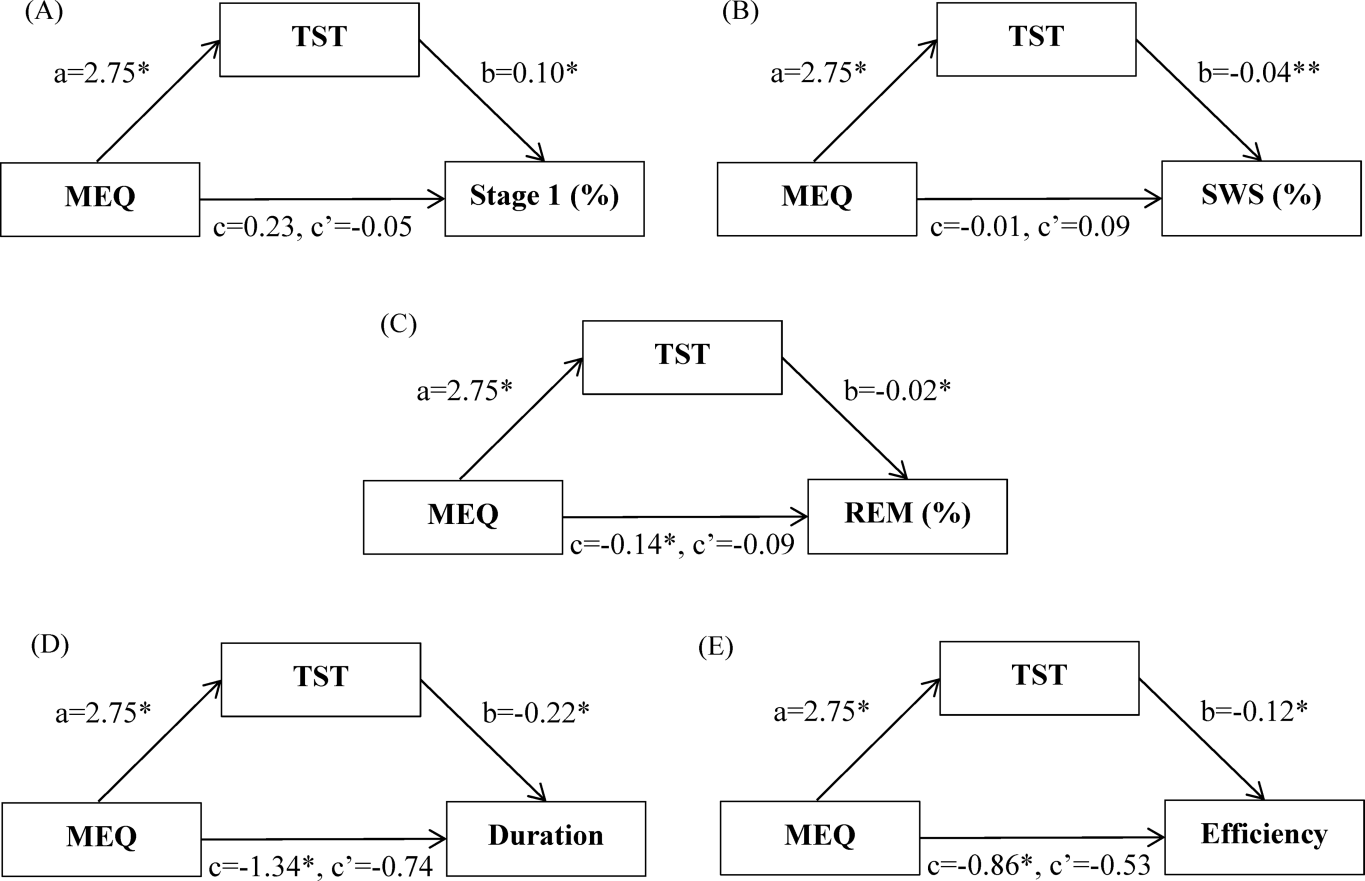
Mediation models of the components of TST on the relationship between MEQ and Stage 1(%), SWS(%), REM(%), sleep duration(min), and sleep efficiency(%) of afternoon nap. (A) TST mediated pathway between MEQ and Stage 1(%); (B) TST mediated pathway between MEQ and SWS(%); (C) TST mediated pathway between MEQ and REM(%); (D) TST mediated pathway between MEQ and sleep duration(min); (E) TST mediated pathway between MEQ and sleep efficiency(%).

Table 3 and Fig 1 show the results of the mediation model used to determine the indirect effects. All regression analyses were performed after adjustment for BMI as the only significant demographic variable (Table 2). We examined the potential mediating effects of nocturnal sleep estimated according to TST on the relationship between the MEQ and each recorded nap parameter. The results indicated that TST mediated the relationship between the MEQ and Stage 1%, SWS%, and REM%, with mediating effects of 0.28 (95% confidence interval [CI]: 0.07–0.79), −0.10 (95% CI: −0.19 to −0.03), and −0.05 (95% CI: −0.12 to −0.00), respectively. Furthermore, TST significantly mediated the association between the MEQ and sleep duration and that between the MEQ and sleep efficiency, with indirect effects of −0.60 (95% CI: −1.44 to −0.13) and −0.32 (95% CI: −0.79 to −0.10), respectively. However, TST did not mediate the relationship between the MEQ and Stage 2% because it was not significantly associated with Stage 2%.

**Table 3:** Mediating model for the effects of TST on the relationship between the MEQ and PSG-recorded naps after adjustment for BMI (N = 52).

Model	Regression test			Indirect effect	Bootstrap with bias-corrected 95% CI
	B	SE	*p*		
TST mediates the MEQ → Stage 1 (%) link					
MEQ → Stage1	0.23	0.33	0.49	0.28	0.07, 0.79
MEQ → TST	2.75	0.95	0.01		
TST → Stage1	0.10	0.05	0.04		
MEQ → Stage1 | TST	−0.05	0.35	0.88		
TST mediates the MEQ → Stage 2 (%) link					
MEQ → Stage 2	0.41	0.34	0.23	NS	
MEQ → TST	2.75	0.95	0.01		
TST → Stage 2	0.07	0.05	0.20		
MEQ → Stage 2 | TST	0.23	0.36	0.53		
TST mediates the MEQ → SWS (%)^+^ link					
MEQ → SWS	−0.01	0.07	0.88	−0.10	−0.19, −0.03
MEQ → TST	2.75	0.95	0.01		
TST → SWS	−0.04	0.01	0.00		
MEQ → SWS | TST	0.09	0.07	0.21		
TST mediates the MEQ → REM (%)^+^ link					
MEQ → REM	−0.14	0.06	0.02	−0.05	−0.12, −0.00
MEQ → TST	2.75	0.95	0.01		
TST → REM	−0.02	0.01	0.04		
MEQ → REM | TST	−0.09	0.06	0.16		
TST mediates the MEQ → Sleep duration (min) link					
MEQ → Sleep duration	−1.34	0.63	0.04	−0.60	−1.44, −0.13
MEQ → TST	2.75	0.95	0.01		
TST → Sleep duration	−0.22	0.09	0.02		
MEQ → Sleep duration | TST	−0.74	0.65	0.26		
TST mediates the MEQ → Sleep efficiency (%) link					
MEQ → Sleep efficiency	−0.86	0.33	0.01	−0.32	−0.79, −0.10
MEQ → TST	2.75	0.95	0.01		
TST → Sleep efficiency	−0.12	0.05	0.02		
MEQ → Sleep efficiency | TST	−0.53	0.34	0.13		

^+ ^Box–Cox transformations with λ_1_ = 0 and λ_2_ = 0.01

B, unstandardized coefficient; SE, standard error; CI, confidence interval; MEQ, morningness–eveningness questionnaire; TST, total sleep time; SWS, slow-wave sleep; REM, rapid eye movement.

## Discussion

According to our review of relevant studies, the present study is the first to specifically examine the mediation effects of nocturnal sleep on the relationship between morningness–eveningness preference and the sleep architecture of naps in university students. The present findings reveal that although the MEQ did not directly correlate with the sleep stage percentage of naps, the effects of the MEQ on Stage 1%, SWS%, REM%, sleep duration, and sleep efficiency of naps were mediated by the TST in the week preceding the study.

The results support our hypothesis that nocturnal sleep mediates the relationship between morningness–eveningness preference and nap quality. As mentioned in Introduction, studies have confirmed the relationship between morningness–eveningness preference and the quality or quantity of nighttime sleep [10, 18] and that increased sleep debt due to nighttime sleep disturbances can result in the tendency to sleep in the daytime [13, 14]. Thus, morningness–eveningness preference may affect nap quality through the mediation of nocturnal sleep. Moreover, SOL and WASO were not tested for mediating effects in this study because only TST significantly correlated with the sleep stage of naps. Vela-Bueno, Fernandez-Mendoza [14] reported similar results of a significant correlation between nap length and total weekend nighttime sleep and a nonsignificant relationship between nap length and nighttime sleep latency.

As shown in Table 3 and Fig 1, TST had negative effects on SWS% and nap duration and the MEQ had positive effects on TST. People with eveningness are more likely to sleep less on weekdays than are those with morningness [10, 18]. Furthermore, significant correlations between nighttime sleep and nap length have been observed [14, 33]. Enhanced SWS following sleep deprivation was correlated with the duration of prior wakefulness [34]. These findings illustrate that the later the participants went to bed, the shorter were their nocturnal sleep durations, resulting in longer sleep durations and SWS of naps to compensate for lost nighttime sleep. These results indicate that the indirect effects of TST were significant between the MEQ and Stage 1%, SWS%, and sleep duration. One previous study reported that the tendency toward later sleep latency was correlated with longer durations of afternoon naps [35] and revealed that adolescents with eveningness had higher sleep requirements than did those with morningness [10]. Vgontzas, Zoumakis [36] reported that modest sleep loss was significantly associated with sleepiness in young men and women. Therefore, a higher SWS% of naps may be a result of spending less time reaching the SWS stage because of daytime sleepiness. These results may be explained simply by high sleep debt and attempts to recover it with daytime sleep.

Table 3 and Fig 1 show the partial mediation effects of TST on the relationship between MEQ and REM% and the sleep efficiency of naps. The MEQ had significantly negative effects on the REM% of naps. This result is consistent with the findings of one previous study, which indicated that REM sleep propensity is affected by the circadian phase of naps [37]. However, according to our understanding, the effects of nocturnal sleep on REM and the sleep efficiency of afternoon naps have not been previously investigated. The present study indicated a negative relationship between TST in the week preceding the study and REM% and sleep efficiency of naps.

As shown in Table 1, BMI was the only significant demographic variable and was considered the control variable in the mediating model (Table 3). One study indicated that overweight and obese participants slept less than participants with healthy BMI levels [38] and shorter sleepers younger than 65 years were associated with higher BMI levels [39]. Another study reported that obese participants had lower sleep efficiency than did controls [40]. In the present study, obesity affected the afternoon naps of young adults.

## Limitations

This study had several limitations. First, we did not select participants according to their chronotypes. Second, we did not restrict the participants’ sleep parameters or collect data on the factors that affected their sleep parameters. Factors such as previous physical activities, architecture of previous nocturnal sleep, or duration of wakefulness in previous nocturnal sleep could be correlated with naps or morningness–eveningness preference. Third, sleeping in an unfamiliar environment and the discomfort of being attached to recording equipment might have affected the measurements, and findings from a single PSG recording could be ambiguous. Moreover, most participants were women and we did not evaluate their menstrual cycles, which may have influenced sleep duration [41].

## Conclusion

Our study examined the mediation effects of nocturnal sleep on the relationship between the morningness–eveningness preference and sleep architecture of naps in university students. The present findings revealed that although the MEQ did not directly correlate with the percentages of nap sleep stages, the effects of the MEQ on Stage 1%, SWS%, REM%, sleep duration, and sleep efficiency of naps were mediated by the TST in the week preceding the study. This preliminary study suggests that nap quality is affected by the morningness–eveningness tendency through the mediation of total nocturnal sleep time. Therefore, future studies should be carefully designed to consider nighttime sleep patterns when analyzing the effects of chronotypes on daytime sleep.
